# A cross-sectional study to assess pragmatic strengths and weaknesses in healthy ageing

**DOI:** 10.1186/s12877-022-03304-z

**Published:** 2022-08-23

**Authors:** Dize Hilviu, Ilaria Gabbatore, Alberto Parola, Francesca M. Bosco

**Affiliations:** 1 GIPSI Research Group, Department of Psychology, University of Turin, Turin, Italy; 2 Research Unit of Logopedics, Faculty of Humanities, University of Oulu, Oulu, Finland; 3 Department of Linguistics, Cognitive Science and Semiotics, Aarhus University, Aarhus, Denmark; 4 Neuroscience Institute of Turin - NIT, University of Turin, Turin, Italy

**Keywords:** Ageing, Pragmatics, Multimodal communication, Decay, Language, Lifespan

## Abstract

**Background:**

Ageing refers to the natural and physiological changes that individuals experience over the years. This process also involves modifications in terms of communicative-pragmatics, namely the ability to convey meanings in social contexts and to interact with other people using various expressive means, such as linguistic, extralinguistic and paralinguistic aspects of communication. Very few studies have provided a complete assessment of communicative-pragmatic performance in healthy ageing.

**Methods:**

The aim of this study was to comprehensively assess communicative-pragmatic ability in three samples of 20 (*N* = 60) healthy adults, each belonging to a different age range (20–40, 65–75, 76–86 years old) and to compare their performance in order to observe any potential changes in their ability to communicate. We also explored the potential role of education and sex on the communicative-pragmatic abilities observed. The three age groups were evaluated with a between-study design by means of the Assessment Battery for Communication (ABaCo), a validated assessment tool characterised by five scales: linguistic, extralinguistic, paralinguistic, contextual and conversational.

**Results:**

The results indicated that the pragmatic ability assessed by the ABaCo is poorer in older participants when compared to the younger ones (main effect of age group: *F*(2,56) = 9.097; *p* < .001). Specifically, significant differences were detected in tasks on the extralinguistic, paralinguistic and contextual scales. Whereas the data highlighted a significant role of education (*F*(1,56) = 4.713; *p* = .034), no sex-related differences were detected.

**Conclusions:**

Our results suggest that the ageing process may also affect communicative-pragmatic ability and a comprehensive assessment of the components of such ability may help to better identify difficulties often experienced by older individuals in their daily life activities.

**Supplementary Information:**

The online version contains supplementary material available at 10.1186/s12877-022-03304-z.

## Background

The literature on senescence suggests the fact that several changes accompany individuals during the ageing process, e.g., changes in physical health [1, 2], mood [3, 4] and cognition [5, 6]. Part of this literature consists of studies on basic aspects of language, such as syntax, morphology, lexicon or vocabulary [7–11]. For instance, empirical research has reported that phonemic and semantic fluency, investigated by asking older adults to pronounce all the words they remember beginning with a specific letter and words belonging to a specific category, start to decrease at 45–50 years of age [12] while crystallized ability measured by vocabulary, seems to remain stable even in early dementia [6, 13].

A fairly neglected area of research in the literature on ageing is that of communicative-pragmatic ability, which refers to the appropriate use of different expressive means, such as linguistic, non-verbal/extralinguistic, e.g., gestures and facial expressions, and paralinguistic, e.g., intonation and prosody, in order to convey a communicative meaning in a specific social context [14, 15]. To the best of our knowledge, only a couple of previous studies have focused on the pragmatic use of language in senescence [16, 17]. Both investigated the linguistic aspect of pragmatic ability in healthy ageing adults using the Right Hemisphere Language Battery (RHLB) [18], and thus examined several facets of pragmatic language, namely the lexical-semantic level, written and visual metaphors, inference, humour, prosody and conversation. Zanini and colleagues [16] used an adapted version of the battery for Italian participants and evaluated individuals divided into six age ranges (from 20 to 79 years old), whereas Daniluk and Borkowska [17] administered a Polish version of the RHLB to participants grouped by age (65 to 70 vs. 71 to 90 years old). The results of both studies highlighted a general decline starting at around 70 years of age; only a few exceptions were detectable, namely prosody in the Italian study and conversation in the Polish one. It is worth noting the samples in the above-mentioned studies were not completely comparable in terms of age. Moreover, as for the different result regarding prosody, this might be due to the use of different tasks. Nevertheless, other studies have reported age-related changes in terms of paralinguistic cues, such as prosody, intonation, rhythm and tone of the voice [19–22].

Besides the investigations referred to above, a few other studies have explored specific facets of pragmatic language ability and observed an age-related difficulty in understanding some specific phenomena such as figurative language (e.g., metaphors and proverbs) [23–26], presuppositions [27], and humour [28].

Within the pragmatic domain, it is also possible to identify changes in non-verbal/extralinguistic means of expression as a consequence of ageing. The literature on this topic reports that the ability to perform and comprehend gestures [29–31] or facial expressions [4] is reduced as a function of age. More specifically, a study suggests that older adults seem to ignore the gesture when it is accompanied by speech and to prefer the linguistic information.

However, despite its importance, the multimodal facet of communicative-pragmatic ability has never been thoroughly investigated in the older population, as demonstrated by the lack of studies investigating non-verbal/extralinguistic and paralinguistic means of communication, and language, at the same time in the same sample of ageing individuals.

In addition, communicative interactions using different expressive means are also based on the ability to adhere to the social situation through the use of contextual and conversational rules [32, 33]. Some studies show that older adults exhibit an alteration of some facets of this ability over the years, such as the production of more verbose and less informative communicative acts compared to younger adults [34–36]. Another study also reported that the ability to discriminate contextually-based communicative acts that are socially appropriate or inappropriate declines with age [37].

All the above-mentioned studies suggest that several age-related declines occur in older adults’ ability to communicate, even if some communicative aspects, like the ability to tell more structurally complex narratives [34, 38] may be preserved or might even ameliorate in late adulthood and then decay in older adults (aged over 75 years). Therefore, it seems that the decay or preservation of communicative-pragmatic ability could be modulated by certain factors, for instance the level of education. Indeed, previous literature has shown that a high level of education is linked to better performance in tasks that require communicative-pragmatic ability [16, 25, 39]. Another moderating factor could be sex, although this is still a debated issue. Indeed, data in the literature show that female participants perform better than male participants in tasks assessing certain skills related to communicative-pragmatic ability, such as verbal fluency [40], emotional prosody [41, 42], and social appropriateness [43]. However, other research does not support these findings and has reported that there are no structural and functional differences between the female vs. the male brain [22, 44]. Finally, recent research provides a new perspective suggesting that, despite the differences between female and male individuals being seemingly very small, such a slight discrepancy in language performance might increase and result in a more significant difference when associated with impairments or pathological conditions [45].

To recap, the studies in the current literature have mainly focused on linguistic aspects (e.g., verbal fluency) of communication, paying less attention to other expressive means, such as extralinguistic and paralinguistic cues; the same applies to the fundamental aspects of pragmatic ability, like coherence during a conversation and appropriateness with respect to the social context. To the best of our knowledge, no previous studies have performed a comprehensive assessment, using a unified protocol, investigating all the above-mentioned aspects at the same time in healthy ageing.

The main purpose of this study was to provide a wide assessment of communicative-pragmatic ability in three age groups: two of older adults (65–75 and 76–86 years old) and one of younger adults (20–40 years old), by mean of a between-study design and using a single validated assessment tool, the Assessment Battery for Communication (ABaCo) [46–49]. ABaCo has been validated for the Italian population [48] (Angeleri et al. 2012) and it has been partially adapted in English [50], Serbian [51] Finnish [52], and Portuguese [53]. The ABaCo is made up of five different evaluation scales—linguistic, extralinguistic, paralinguistic, context, and conversational—investigating participants’ communicative-pragmatic ability. The ABaCo has shown in previous research to be a valid tool for investigating communicative-pragmatic ability in patients with traumatic brain injury [54–59], schizophrenia [60–65], left and right acquired brain lesions [66, 67]. We expected to observe age-related differences on the pragmatic performance on the ABaCo in the three groups of participants, from the oldest (poorest performance) to the youngest group (best performance). More in detail, we investigated whether such difference in pragmatic performance applied for each of the scales investigated, i.e., linguistic, extralinguistic, paralinguistic, contextual and conversational. We also explored the possible role of participants’ educational level in explaining their pragmatic performance.

Finally, as the impact of sex on pragmatic ability is not yet totally clear, we decided to explore whether any sex-related differences could be observed in participants’ communicative-pragmatic performance as measured on the ABaCo scales.

## Methods

### Participants

Sixty participants were enrolled for the present study and divided equally into three age groups: *Young Adults*, YA, between 20 and 40 years; *Older Adults*, OA, with ages ranging from 65 to 75 years and *Senior-Older Adults*, SOA, with ages ranging from 76 to 86 years. All demographic details (i.e., age, sex, and education) have been collected in Table 1. The three groups matched for sex (10 female participants and 10 male participants for each age group) and educational level (*F* (2,57) = 2.431; *p* = 0.097). Participants were recruited through different modalities: Universities of the Third Age, local social clubs, local voluntary and charitable associations, cultural events, and personal contacts. All participants were native Italian speakers.

**Table 1:** Descriptive data for demographic information of each age group

	Age	Sex	Education
	Mean (SD)	(F;M)	Mean (SD)
YA	29.55 (5.80)	10;10	13.50 (3.65)
OA	70.30 (3.06)	10;10	10.80 (4.70)
SOA	80.90 (2.81)	10;10	10.80 (4.96)
Total	60.25 (22.70)	30;30	11.70 (4.58)

*Note.* Young Adults (20–40 years old), Older Adults (65–75 years old) and Senior-Older Adults (76–86 years old). *SD* Standard deviation, *F* Female participants, *M* Male participants

Participation was voluntary and potential participants demonstrating interest in the research were asked to carefully read the informative flyer of the study, reporting inclusion criteria: presence of severe cognitive or linguistic deficits, evidence of current or past neurological disorder (e.g., epilepsy), substance or alcohol use disorder, anamnesis of major neurological or neuropsychological disease, hearing or vision problems, history of head injury and consumption of mood stabilizers. The flyers specified that potential participants were asked to take part in the study only in absence of all the above-mentioned exclusion criteria. Otherwise, no data were collected. The only data we collected refer to one participant, excluded after a preliminary evaluation because of a pharmacological condition that was not detected by the aforementioned criteria. If none of the exclusion criteria was violated, only subjects with sufficient cognitive and linguistic skills, as resulting from the achievement of a cut-off score on the Montreal Cognitive Assessment (MoCA: ≥ 19.5) [68], a short form of the Token Test (TT: ≥ 4.5) [69] and the Naming task of the Aachener Aphasie Test (AAT: ≥ 108) [70] were included in the sample. Among the volunteers who, in absence of exclusion criteria, were administered the screening tests, two did not achieve the cut-off scores (AAT and TT).

#### Statistical power

We calculated statistical power using G*Power 3.1.9.7 software [71]. The normative data for the ABaCo [48] report group differences (Cohen’s d) for the age ranges considered in the present study (15–34 and 55–75) between 0.50 and 0.70. Since our design involves 3 groups (YA, OA, and SOA), 5 repeated-measures (i.e., ABaCo subscales) and 1 covariate (i.e., education), by conservatively assuming an effect size of 0.3 (smaller than expected considered that the age range of oldest adults in the present study is higher, i.e., 76–86, compared to the oldest age range of the ABaCo normative values, i.e., 55–75), the estimated sample size for reaching a statistical power ≥ 0.95 is 60 participants. The post-hoc observed power calculated by using IBM SPSS Statistics 26 software SPSS confirmed that the statistical power for the main effects and interactions is ≥ 0.95.

### Material

Participants’ pragmatic ability was evaluated with the Assessment Battery for Communication (ABaCo), a manualized and validated tool that investigates different facets of pragmatic ability [46–49]. The battery includes five scales: linguistic, extralinguistic, paralinguistic, contextual and conversational. The whole battery comprises 72 items in the form of live interactions with the experimenter and 100 short video clips (20–25 s each), followed by specific open questions for each item (for a total of 90 min). The ABaCo has shown good psychometric properties in terms of internal consistency within each scale (Cronbach alpha: 0.52 < α < 0.91), inter-rater reliability (Cohen’s kappa: k = 0.76 to k = 0.96) as well as content and construct validity [47]. The normative values are available from 16 to 75 years of age, stratified for age and education, and the tool has already been used in several empirical studies [54, 63, 66, 67, 72, 73]. A brief description of the ABaCo scales is provided in the following section  [47–49, 67].

#### Linguistic and extralinguistic scales

Both scales evaluate the ability to understand and produce basic communicative acts, i.e., statements, questions, commands and requests [74], direct and indirect communicative acts [75]. The Linguistic scale evaluates items using the linguistic channel while the Extralinguistic scale assesses items using extralinguistic means of expression, e.g., gestures or body movements.

#### Paralinguistic scale

The Paralinguistic scale assesses the ability to understand and produce all aspects that connote communicative acts, ascribing an emotional nuance to communication, e.g., through prosody and intonation. The items that make up this scale evaluate emotions, paralinguistic inconsistencies and basic communicative acts.

#### Contextual scale

The Contextual scale assesses the ability to understand and produce appropriate communicative acts with respect to the social and conversational context of the situation, through the respect of discourse rules (Grice’s maxims) and social compliance.

#### Conversational scale

The Conversational scale verifies participants’ ability to have a face-to-face conversation with the examiner. The scale measures turn-taking ability, the capacity to stick to the topic and the ability to enrich and extend the discussion by providing new ideas.

### Administration and Coding Procedure

Three master’s students in Psychology tested participants individually—at home—in two sessions each lasting one hour. Each examiner tested the same number of participants (n = 20) equally distributed among the three experimental groups (YA, OA, SOA). The ABaCo video clips were presented on a laptop, while the experimenter and the participants sat at a table facing each other.

The sessions were video recorded and two independent judges, master’ students in Psychology who had recently successfully passed an exam in Psychology of Communication, examined the video recordings and coded participants’ performance off-line, based on the coding system described in the ABaCo administration manual [46]. All items were scored either as correct (1 point) or incorrect (0 points), based on the coding manual [46]. See Additional file 1 for examples of correct and incorrect answer. The final score for each scale and ABaCo total is calculated as the mean of all items’ scores (therefore, the final score can vary between 0 and 1). The degree of reliability between the two judges was calculated using 45% of the sample and the average intraclass correlation coefficient (ICC) measure was 0.808 with a 95% confidence interval from 0.776 to 0.836 (*p* = 0.000). The administrators and coders received specific training before the start of the study, to ensure the use of homogeneous procedures and compliance with the administration and coding rules described in the ABaCo manual. All participants signed the informed consent form and gave their written permission. They were also informed of the possibility of interrupting the test at any time. The Bio-Ethical Committee of the University of Turin approved the study.

### Data analysis

#### Age-related differences

Analyses were performed using the IBM SPSS Statistics 26 software. Participants’ ABaCo scores were submitted to a 3 × 5 repeated measures analysis of covariance (ANCOVA) with Age group (YA, OA, and SOA) as the *between-subjects factor* and Scales (linguistic, extralinguistic, paralinguistic, contextual and conversational) as the *within-subjects factor*. Linear contrast analysis was performed with the overall pragmatic performance (ABaCo total score). Pairwise post-hoc comparisons were calculated for significant effects and interactions with Bonferroni correction. Education was added as a covariate in order to control for the role of this variable.

#### Sex-related differences

In order to explore potential sex-related differences, scores of female and male participants, on each scale and in each age group, were compared using independent samples *t*-tests.

## Results

### Results of age-related differences

Repeated measures ANCOVA revealed a main effect of Age group—YA, OA and SOA—(*F*(2,56) = 9.097; *p* < 0.001; η^2^_p_ = 0.245) and Scales—Linguistic, Extralinguistic, Paralinguistic, Contextual and Conversational, i.e., all the scales composing the ABaCo—(*F*(4,224) = 20.084; *p* < 0.001; η^2^_p_ = 0.264). The interaction Age group*Scales was also significant (*F*(8,224) = 2.335; *p* = 0.020; η^2^_p_ = 0.077).

The significant linear contrast (*F*(1,57) = 20.169; *p* < 0.001), indicates a linear decrease in performance from YA to SOA. Bonferroni-corrected pairwise comparisons showed that overall pragmatic performance on the ABaCo (all scales pooled) decreased with age. The YA performed significantly better than the OA group (*p* = 0.003) and the SOA group (*p* = 0.001). By contrast, no significant difference was observed between the two groups of older participants in terms of their performance on the ABaCo scales (*p* = 1.000), see Table 2.

**Table 2:** Descriptive results for each Scale of the ABaCo, and ABaCo total score, in each age group

Scales	Young Adults	Older Adults	Senior-Older Adults
	Mean (SD)	Mean (SD)	Mean (SD)
Linguistic	.87 (.09)	.80 (0.10)	.80 (.07)
Extralinguistic	.80 (.10)	.64 (0.12)	.65 (.15)
Paralinguistic	.88 (.08)	.77 (0.12)	.74 (.13)
Contextual	.93 (.08)	.85 (0.14)	.82 (.09)
Conversational	.99 (.03)	.99 (0.06)	.97 (.09)
ABaCo total score	.87 (.07)	.76 (0.09)	.76 (.08)

*Note.* Young Adults (20–40 years old), Older Adults (65–75 years old) and Senior-Older Adults (76–86 years old). *SD* Standard deviation

**Fig. 1:**
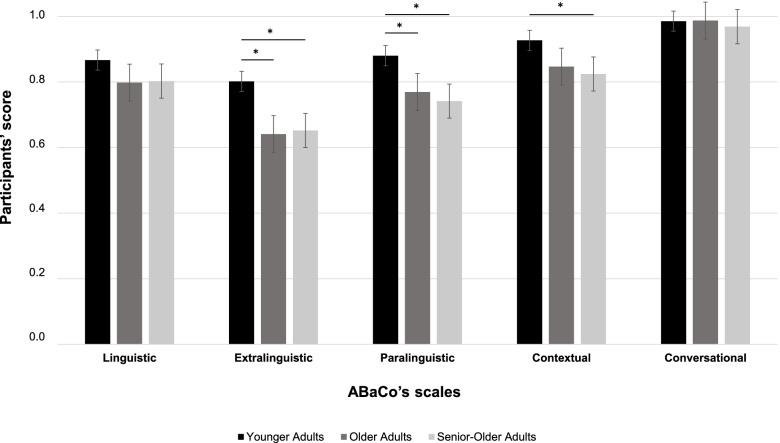
Participants’ mean scores by age group on each Scale of the ABaCo. *Note.* Error bars: standard error

Considering the Scales factor, (with all age groups pooled), the results showed that the Linguistic scale differed significantly from the Extralinguistic (*p* = 0.000), Contextual (*p* = 0.037) and Conversational (*p* = 0.000) scales. Moreover, significant differences were also observed between the Extralinguistic scale and the Paralinguistic (*p* = 0.000), Contextual (*p* = 0.000) and Conversational (*p* = 0.000) scales. The differences between the Paralinguistic and Contextual (*p* = 0.001) and Conversational (*p* = 0.000) scales were also significant. Finally, the Contextual and Conversational scales were also found to be significantly different (*p* = 0.000). The Extralinguistic scale was reported as the most difficult, as shown by the participants’ scores, followed by the Paralinguistic and Linguistic scales. The Contextual scale was found to be the easiest, followed by the Conversational scale (see Fig. 1).

Finally, Bonferroni-corrected pairwise comparisons for the interaction Scales*Age group highlighted significant differences between the YA and the two groups of older individuals on the Extralinguistic scale (*p* < 0.005), on the Paralinguistic scale (*p* < 0.047) and on the Contextual scale between the YA group and the OA group (*p* = 0.013), see Fig. 1.

No differences were found among groups on the other scales.

These effects were significant even after controlling for the role of the covariate Education, which was found to be significant (*F*(1,56) = 4.713; *p* = 0.034; η^2^_p_ = 0.078) suggesting a direct role of education in participants’ overall pragmatic ability (all groups, i.e., YA, OA and SOA, pooled together).

### Results of sex-related differences

The results of the independent sample *t*-tests are presented in Table 3. The analysis of the results revealed no significant differences when comparing female and male participants on the different scales of the ABaCo in the three age groups.

**Table 3:** Descriptive results of female and male participants on the ABaCo’s scales, and ABACo total score, and results of t-tests

	F	M	df	*t*	*p*-value
	Mean (SD)	Mean (SD)			
**Linguistic**					
YA	.89 (.09)	.85(.10)	18	1.055	.305
OA	.79 (.08)	.81 (.12)	18	-.501	.622
SOA	.82 (.04)	.78 (.09)	18	1.243	.230
**Extralinguistic**					
YA	.80 (.09)	.80 (.11)	18	.009	.993
OA	.61 (.09)	.68 (.15)	18	-1.243	.230
SOA	.64 (.20)	.67 (.09)	18	-.434	.670
**Paralinguistic**					
YA	.89 (.05)	.87 (.09)	18	.782	.444
OA	.74 (.08)	.80 (.15)	18	-.976	.342
SOA^a^	.79 (.17)	.70 (.06)	11.374	1.587	.140
**Contextual**					
YA	.95 (.06)	.90 (.09)	18	1.407	.177
OA	.85 (.08)	.84 (.18)	18	.181	.859
SOA	.86 (.07)	.78 (.10)	18	2.077	.052
**Conversational**					
YA	.99 (.03)	.98 (.04)	18	.896	.382
OA^a^	1.00 (.00)	.98 (.08)	9.000	1.000	.343
SOA	.98 (.07)	.96 (.10)	18	.545	.592
ABaCo total score					
YA	.88 (.04)	.85 (.08)	18	1.020	.321
OA^a^	.75 (.06)	.78 (.12)	13.831	-.790	.443
SOA^a^	.78 (.10)	.73 (.04)	11.027	1.315	.215

*Note.* Mean and standard deviations of the performance of female and male participants of the three groups on the five scales of ABaCo. *F* Female participants, *M* Male participants, *YA* Young Adults (20 – 40 years old); *OA* Older Adults (65 – 75 years old); *SOA* Senior-Older Adults (76 – 86 years old)

^a^ Welch’s t-test for unequal variances was used instead of Student’s t-tests

## Discussion

The aim of the present research was to comprehensively assess communicative-pragmatic ability in three groups of adults belonging to different age ranges (YA: 20–40, OA: 65–75, and SOA: 76–86 years old) and to compare their performance. For the evaluation we used the Assessment Battery for Communication which is a validated clinical tool able to detect specific changes that occur in different clinical conditions [46–49].

Considered as a whole, our results showed that the two groups of older individuals performed worse than the group of younger participants. These findings will now be discussed more in detail by considering each scale separately.

Firstly, a substantial difference in ability was observed on the extralinguistic scale, which was also reported to be the most difficult one since it emerged as the scale with the lowest scores. Items belonging to this scale are designed to evaluate communicative acts using gestures or body movements. Individuals in both the OA and SOA groups exhibited poorer performance compared to the group of younger adults. This outcome is perfectly in line with previous studies that assessed the impact of age on extralinguistic ability [4, 29–31, 76, 77]. For instance, Cocks and colleagues [76] investigated the integration of iconic gestures and speech in two groups of participants in different age-groups—22–30 and 60–76 years old—and they found that individuals in the second group performed less well in tasks assessing the comprehension of extralinguistic cues. The authors of a recent study [78] proposed a cognitive explanation for this decline, and investigated whether the age-related decline in the production of gestures is linked to working memory ability or mental imagery skills. They compared the scores obtained by a group of older individuals to those of a group of younger persons in tasks involving the use of gestures, and found that mental imagery skills were associated with age-related changes in representational gesture production [78]. Therefore, the difference in extralinguistic ability appears to be partially related to a decay in visual-spatial skills [77, 78]. However, this reduction seems to progress slowly since, in our study, no difference was detected between the two groups of older individuals.

Secondly, significant differences were also detected on the paralinguistic scale, which assesses the use of several cues, such as prosody, intonation and rhythm, used to enrich content expressed through linguistic and extralinguistic means. The two groups of healthy older participants obtained significantly lower scores than the group of younger individuals. This finding is in line with previous research that focused on affective prosody and intonation in ageing [17, 79, 80]. For instance, Orbelo and colleagues [81] examined affective prosody, i.e., the ability to express emotions via prosodic cues, such as the tone of the voice. The participants were divided into three groups—in which the average age was 30, 55 and 74 years – and were asked to repeat or to produce spontaneous narrations by discussing emotional events in their personal lives. Their results showed that, while production turned out to be unimpaired, the group of older individuals had greater difficulty with the comprehension of affective prosody, as compared to the two other age groups. The authors concluded that, although the individuals in the oldest group did not have any specific hearing deficits, the natural and physiological process of ageing could have affected their ability to discriminate pitch variations. Older adults identify the presentation of emotions, such as sadness, less accurately, even when conveyed through speech, as compared to younger adults [82]. Further literature suggests that the decline in the ability to recognize affective prosody, that characterizes older individuals, could be the result of neural modifications that occur in the right hemisphere with the ageing process [83, 84].

The third area in which a significant difference was observed between groups was assessed by the contextual scale, which evaluates the appropriate use of communicative behaviours with respect to the specific social context in which the communicative interaction takes place. In this case, we found a significant difference in performance between the SOA group and the YA but not between the OA group and the YA. The difference is in line with previous results [37, 85, 86], some of which suggest that this decline in social appropriateness could be related to a decay in executive functioning [85, 86]. The similar level of performance between the OA group and the YA could suggest that the difficulties in terms of social appropriateness as assessed by the contextual scale of the ABaCo only arise in later stages of the ageing process, and they are thus specific to the oldest group.

As for the linguistic scale, assessing the comprehension and production of various communicative acts (i.e., basic speech acts, direct and indirect speech acts, irony and deceit) principally conveyed through linguistic means, we did not detect any significant difference among the groups. Specifically, the results regarding this scale showed that despite the scores obtained by the group of younger individuals being slightly higher than those of the two groups of older individuals, no significant difference was found. This result is not surprising, since the linguistic modality represents the most used expressive means. Furthermore, our outcome is in line with previous research that highlighted how some aspects of linguistic ability are preserved in ageing while others may decline [87]. However, further research is needed in order to understand which specific aspects of pragmatic language ability may be preserved, such as the ability to manage simple acts, and which may decline, e.g., the ability to handle more complex acts like irony.

Furthermore, we did not observe any difference among the groups in terms of performance on the conversational scale, which assesses the ability to maintain a conversation and respect conversational topics and turn taking. This outcome is in line with the result obtained by Daniluk and Borkowska [17] but in contrast with other studies where an age-related decline was detected [88, 89]. In a study by Pereira and collaborators [90], the conversational discourse of a group of older individuals (70 years old) was transcribed, analysed and compared to the transcription of conversational samples of a group of young adults (27 years old). The results highlighted the difficulty of older adults to respect turn taking, to maintain the topic of the conversation and to return to the original topic after a thematic shift. When considering such inconsistency, it seems important to recall that in our conversational task, participants were engaged in a semi-spontaneous conversation with the experimenter in a quiet setting and, as suggested by a previous study, that age-related differences in conversation are minimized when background noise is reduced, and that this also leads to better comprehension in older adults [91].

Finally, the significant effect of education is consistent with previous studies which pointed out that educational level could have an impact on communicative performance [16, 25]. Detecting a role of education in pragmatic ability is not surprising, since education represents one of the main components of the Cognitive Reserve, which refers to a broad combination of experiences a person has throughout life, including access to education, having a prominent role in determining the early impact of degenerative diseases [92]. Therefore, a higher level of education may have an important role in compensating for a potential cognitive decline that occurs with ageing [39, 93].

By contrast, we did not find any significant effect of sex on pragmatic ability, having observed comparable performance between female and male participants in the different age groups. Our results, thus, support those of previous studies in which no difference in language performance was found between female and male participants [22, 44].

This study has some limitations: firstly, increasing the sample size might highlight greater variability, especially when considering the current preliminary investigation on sex-related differences. A larger sample could, indeed, have enabled us to obtain further details concerning age-related and also sex-related changes in communicative-pragmatic performance. Secondly, for the assessment of the decline in communicative-pragmatic ability, a longitudinal analysis might be useful, as it would allow to follow the progression and evolution of this ability in the same sample of participants. This might be the subject of further investigation in the near future, having at least part of this sample of participants as a starting point. Moreover, we believe that forthcoming research should focus on how the decline in communicative-pragmatic ability occurs, since several changes are associated with ageing, namely changes in perceptual sensitivity [88, 91], Executive Functions (EF), i.e., cognitive processes that allow individuals to perform everyday tasks [6, 94–99] and Theory of Mind (ToM), i.e., the ability to attribute mental states to one’s self and to others [100–103].

## Conclusions

To conclude, communicative-pragmatic ability is essential because it allows individuals to communicate effectively and to perform their activities in everyday life. The deterioration of such ability may contribute to the social isolation that often affects not only pathological, but also healthy older individuals [104]. However, previous studies primarily focused on the linguistic aspects of communication, leaving aside pragmatic ability. In such perspective, our study fills a gap in the literature on ageing because it examines all components of pragmatic expression in two different old age ranges. Hence, our results could form the basis for communicative-pragmatic training activities designed to maintain or strengthen this ability [93, 105] and for the development of assistance systems for the elderly [106].

## Supplementary Information

**Additional file 1. **Examples of items, possible answers and scores from the Assessment Battery for Communication (ABaCo), administrated to participants to evaluate their communicative-pragmatic ability.

## Data Availability

The data are not publicly available for privacy or ethical restrictions.

## Abbreviations

AAT: Aachener Aphasie Test
ABaCo: Assessment Battery for Communication
ANCOVA: Analysis of covariance
EF: Executive Functions
ICC: Intraclass correlation coefficient
M: Mean
MoCA: Montreal Cognitive Assessment
OA: Older Adults
RHLB: Right Hemisphere Language Battery
SOA: Senior-Older Adults
SD: Standard Deviation
ToM: Theory of Mind
TT: Token Test
YA: Young Adults
