# Supplementary material for: A cross-sectional study to assess pragmatic strengths and weaknesses in healthy ageing
Source: BMC Geriatr. 2022 Aug 23;22:699. doi: 10.1186/s12877-022-03304-z (PMC9400309; doi:10.1186/s12877-022-03304-z)
Supplement: Supplementary file 1 — Additional file 1. Examples of items, possible answers and scores from the Assessment Battery for Communication (ABaCo), administrated to participants to evaluate their communicative-pragmatic ability. [file 12877_2022_3304_MOESM1_ESM.docx]

| **Item** | **Test Question** | **Participant’s answer** | **Score** |
| --- | --- | --- | --- |
| **Linguistic Scale** | | | |
| ***Comprehension*** | | | |
| *[L28] – Irony (videoclip)*  Mom is at the sewing machine, while Ermanno is trying his trousers, clearly having different lengths in the two legs. The mom is busy with sewing and not looking and asks: ‘So, are the trousers ok?’. Ermanno replies: ‘For a gala dinner they will be great…!’ | (a) What did the boy say?  (b) Was he serious?  (c) Why did he reply this way to his mom? | (a) They are not fine at all  (b) I don’t know  (c) make her happy | 0 |
| ***Production*** | | | |
| *[L53] – Irony (videoclip)*  Fabio and Claudia are having breakfast. Fabio is staring at the TV and does not realize he has put his elbow on the jam. Claudia looks at him and smiles, until the moment Fabio says – still staring at the TV – ‘Could you please pass me the jam’? | (a) What could the girl answer so to be funny, ironic? | (b) You are eating the jam with the elbow, already! | 1 |
| **Extralinguistic Scale** | | | |
| ***Comprehension*** | | | |
| *[X12] – Basic Speech Acts: Request (videoclip)*  The girl makes some dancing around the room and – looking at the participant -makes a gesture as to say ‘Come and dance with me’ | (a) What did the girl say? | (a) That she is happy | 0 |
| ***Production*** | | | |
| *[X46] – Standard (direct and indirect) communicative acts (videoclip)*  Diego is sitting on the sofa, reading a book. Manuela enters the room, gets close to Diego and makes an interrogative gesture, as to ask: ‘How is the book? Is it good’? | (a) The girl asks how is the book. What gesture can the boy use? | (a) “Thumb up” and appropriate facial expression, as to say “not bad at all!” | 1 |
| **Paralinguistic Scale** | | | |
| ***Comprehension*** | | | |
| *[P18] – Paralinguistic contradiction (videoclip)*  It’s Robert’s birthday, and Paola gives  him a gift saying: ‘Happy birthday!’  Robert unwraps the gift and discovers an  awful tie. With an annoyed expression,  he says: ‘Thanks, really, I needed one of those…’ | (a) What did the boy say?  (b) In your opinion, did the boy like the tie? | (a) The present was not to his taste  (b) no | 1 |
| ***Production*** | | | |
| *[P25] – Emotion expression*  I’m going to ask you to say some sentences. You must say them the way I tell you: | Ask me what time it is. Do it as if you were bored. | ‘What time is it’ with  a neutral tone of voice. | 0 |
| **Context Scale** | | | |
| ***Comprehension*** | | | |
| *[C3] – Grice maxim (videoclip)*  Monica is sitting on the bed, folding some stuff. Federico arrives and asks: ‘Where did you go on holiday?’. Monica replies: ‘Last summer I’ve spent one month on the moon’ | (a) In your opinion, is the reply fine?  (b) Why? | (a) No  (b) She didn’t want to share her own business | 0 |
| ***Production*** | | | |
| *[P3] – Social Norms*  I’m going to ask you to say some sentences: | Imagine you are late for an appointment with your lawyer, and you have to apologize. How  do you apologize? | Good morning, sir, I’m so sorry for the delay. I had an inconvenient. Hopefully you can still meet me. | 1 |
| **Conversational Scale** | | | |
| *[Conv1 – Conv2] – Free conversation*  The examiner engages the participant in four brief conversations about her/his:  (1) Free time  (2) TV  (3) Holidays  (4) Own town  For each conversation, the rater assigns a  score based on participant’s topic and  turn-taking maintenance. |  |  |  |
